# What makes the Asian bush mosquito *Aedes japonicus japonicus* feel comfortable in Germany? A fuzzy modelling approach

**DOI:** 10.1186/s13071-019-3368-0

**Published:** 2019-03-14

**Authors:** Antje Kerkow, Ralf Wieland, Marcel B. Koban, Franz Hölker, Jonathan M. Jeschke, Doreen Werner, Helge Kampen

**Affiliations:** 1 Leibniz Centre for Agricultural Landscape Research (ZALF), Eberswalder Str. 84, 15374 Müncheberg, Germany; 2 Department of Biology, Chemistry, Pharmacy, Institute of Biology, Freie Universität Berlin, Königin-Luise-Str. 1–3, 14195 Berlin, Germany; 3 Leibniz-Institute of Freshwater Ecology and Inland Fisheries (IGB), Müggelseedamm 310, 12587 Berlin, Germany; 4 Berlin-Brandenburg Institute of Advanced Biodiversity Research (BBIB), Altensteinstr. 34, 14195 Berlin, Germany; 5 Friedrich-Loeffler-Institut (FLI), Federal Research Institute for Animal Health, Südufer 10, 17493 Greifswald, Insel Riems Germany

**Keywords:** Asian rock pool mosquito, Biological invasions, Climate change, Landscape interactions, Land use, Machine learning, Nested approach, Species distribution models, Wind speed

## Abstract

**Background:**

The Asian bush mosquito *Aedes japonicus japonicus* is an invasive species native to East Asia and has become established in North America and Europe. On both continents, the species has spread over wide areas. Since it is a potential vector of human and livestock pathogens, distribution and dissemination maps are urgently needed to implement targeted surveillance and control in case of disease outbreaks. Previous distribution models for Europe and Germany in particular focused on climate data. Until now, effects of other environmental variables such as land use and wind remained unconsidered.

**Results:**

In order to better explain the distribution pattern of *Ae. j. japonicus* in Germany at a regional level, we have developed a nested approach that allows for the combination of data derived from (i) a climate model based on a machine-learning approach; (ii) a landscape model developed by means of ecological expert knowledge; and (iii) wind speed data. The approach is based on the fuzzy modelling technique that enables to precisely define the interactions between the three factors and additionally considers uncertainties with regard to the acceptance of certain environmental conditions. The model combines different spatial resolutions of data for Germany and achieves a much higher degree of accuracy than previous published distribution models. Our results reveal that a well-suited landscape structure can even facilitate the occurrence of *Ae. j. japonicus* in a climatically unsuitable region. *Vice versa*, unsuitable land use types such as agricultural landscapes and coniferous forests reduce the occurrence probability in climatically suitable regions.

**Conclusions:**

The approach has significantly improved existing distribution models of *Ae. j. japonicus* for the area of Germany. We generated distribution maps with a resolution of 100 × 100 m that can serve as a basis for the design of control measures. All model input data and scripts are open source and freely available, so that the model can easily be applied to other countries or, more generally, to other species.

## Background

The Asian bush mosquito *Aedes japonicus japonicus* (Theobald, 1901) (Diptera: Culicidae), native to Japan, Korea, Taiwan, China and south-eastern Russia [1], is an invasive species of currently great importance in the northern hemisphere, especially within temperate climatic areas, although it has also been discovered in subtropical and tropical regions such as Florida and Hawaii [2–6]. The first record of the species in Europe was in France in 2000. Since then, it has been detected in 12 European countries [7–10]. In Germany, the species was discovered in 2008 at the Swiss border [11]. Meanwhile, *Ae. j. japonicus* is widespread in the country. Its main areas of distribution are in the west, southwest and southeast [8].

Although it has been assumed that it will be no longer possible to eliminate *Ae. j. japonicus* completely from Germany by means of control measures [8], its continuing spread is closely monitored because the species is a potential vector of disease agents of humans and livestock. Its vector competence includes at least 11 different viruses (including West Nile virus and Zika virus) and two filarial nematode species [12–14].

The aim of this study is to use modelling and the integration of ecological expert knowledge to generate maps that show the risk of colonisation with *Ae. j. japonicus* in Germany as accurately and detailed as possible. The maps should (i) be suitable for informing the public to support preventive measures and initiate targeted control measures in the event of a disease outbreak for whose agent *Ae. j. japonicus* is a competent vector and (ii) assess how the species will spread under the influence of climate change.

Distribution models for *Ae. j. japonicus* already exist for Europe [15] and, more specifically, for Germany [16, 17] and Slovenia [7]. They rely on climate data such as precipitation sums and average monthly and seasonal temperatures with a resolution of 1 × 1 km to 10 × 10 km, and partly on elevation data [7]. As these distribution models score well in their validation, it can be concluded that climatic factors are relevant for the species to become established in a region. However, the distribution maps derived from climate models are not suitable for planning concrete control measures due to their usually broad scale. It should also be noted that recently introduced species may not yet be in equilibrium with their environment, so that the ecological niche determined by machine learning is often calculated too narrowly [15]. In addition, the occurrence of mosquitoes is strongly dependent on local weather events, which can sometimes deviate dramatically from average climatic conditions.

On a smaller scale, the occurrence of certain mosquito species can be predicted using landscape data (see for example [18–20]). Landscape data are an indicator of the occurrence of breeding sites for which each mosquito species has its own requirements. Egg deposition and larval development of the Asian bush mosquito take place in small containers, both in natural habitats, such as stream rock pools and tree holes of deciduous trees [1], and in artificial containers like plant dishes, rainwater catchments and trash cans, the latter particularly often being available in human settlements [11, 21–23]. The landscape does not only affect egg deposition and larval development. It can, for instance, also be correlated with the occurrence of blood hosts and predators. Besides climate and landscape (including land use and further landscape elements), we found that regional mean wind speeds also seem to have an important influence on the presence of *Ae. j. japonicus*.

Based on the assumption that the three factors “climate”, “landscape” and “wind” mainly determine the possible distribution of the species, the question arises with which model type the interaction of the corresponding geodata can be expressed. Requirements for implementation were that (i) it is comprehensible to biologists; (ii) it allows uncertainties; and (iii) the interplay of the factors can be controlled by the modeller. The first point is important as ecological knowledge about *Ae. j. japonicus* is incomplete. As soon as new results from ecological studies emerge, the model can be adapted. The second requirement results from the fact that biological expert knowledge is often expressed by use of linguistic terms instead of exact numbers, which in turn is mainly due to the fact that individuals within a species show a variability with regard to the acceptance of certain environmental conditions.

The fuzzy modelling technique meets all the mentioned criteria. It is a white box modelling approach that allows for the integration of biological expert knowledge [24] and enables the influence of each input variable on the model to be tracked and easily understood by biologists without an informatics background. The fuzzy approach [25] can deal with uncertainties and is ideal for habitat models, as its basic idea is that assignments do not always have to follow Boolean principles, but that there is often a degree of membership. For a habitat model, each environmental variable that is relevant to the species can be divided into fuzzy sets, which are given a name, a so-called linguistic term. For example, if the environmental variable is “wind speed”, it could be divided into the fuzzy sets “comfortable” (unrestricted flight capability), “high” (causing moderate flight restrictions) and “too high” (causing strong flight restrictions). By means of membership functions, values are assigned to the sets with every value having degrees of membership to the sets on a percentage scale. The interplay of environmental variables and their different states can be directly controlled by the modeller with the help of rules.

There are already numerous studies on the ecology of the Asian bush mosquito available, and fuzzy modelling is an established method in ecological niche modelling (see e.g. [26–28]). Our particular research questions were therefore (i) whether fuzzy modelling allows to combine models developed on the basis of machine learning (a climate model), expert knowledge (a landscape model) and additional important data (wind) in such a way that more accurate predictions can be achieved compared to the initial models (landscape only and climate only), and (ii) whether, despite the originally different resolutions of the input data (100 × 100 m, 200 × 200 m and 1000 × 1000 m), the outcome, calculated for the finest scale, performs better in the validation than that of the most detailed input model (landscape). As the novelty of the approach is the combination of local landscape and wind data with large scale mean climate data by means of fuzzy logic, as well as the interplay of ecological expert knowledge and the power of machine learning, we call the approach hereafter a nested approach.

## Methods

### Habitat requirements and selection of model input parameters

For the selection of input data of the fuzzy model, and especially for the development of the landscape model on which the fuzzy model is partly based, both ecological characteristics of the species as well as generally favourable conditions for the occurrence of mosquitoes were considered. To improve our understanding of the species and its potential habitats in the study area we reviewed the literature, talked to other mosquito specialists and statistically assessed various geodata and satellite images of Germany. A summary of the habitat requirements is presented here in order to understand the setup of the model.

Habitat choice of mosquitoes is basically driven by the availability of suitable breeding sites for egg deposition and larval development. *Ae. j. japonicus* uses small breeding habitats and naturally occurs in stream rock pools, kinked bamboo trunks and tree holes of deciduous trees [1]. It can also be found in human settlements, where the larvae develop in small artificial containers, including plant dishes, buckets, trash cans, discarded snack bags, rainwater catchments, fountains and used tires [11, 21–23]. Shade is also beneficial for both larvae and adults of the species as it minimises the risk of breeding site evaporation and desiccation and provides resting places during hot days [2, 22, 29]. The general availability of plants, flowers and fruits is important for mosquito adults, as they feed on plant juices and nectar. Organic material such as leaf litter and pollen is equally important for the larvae, as they feed on detritus and bacteria [22, 30, 31]. For egg production, female mosquitoes need proteinaceous blood meals. *Ae. j. japonicus* females were observed to feed on mammals (such as white-tailed deer, fallow deer, horses and humans) and birds, but not on amphibians or reptilians [11, 32, 33].

Regarding the parameter terrain height, we find a negative correlation of *Ae. j. japonicus* occurrence with height by intersecting collection data with an elevation map (25 × 25 m resolution) and by considering small areas (about 10 × 10 km), which confirms findings of a study in Japan [29]. It seems that *Ae. j. japonicus* prefers valleys over higher altitudes. When looking at the area of Germany, however, there is no relationship between elevation and the occurrence of the mosquito. As land use and climate, which sometimes correlate with height, did not explain the observed distribution pattern, we suspect that the correlations for smaller areas are rather due to wind speed.

To our knowledge, no study exists about how the behaviour and distribution of *Ae. j. japonicus* are affected by wind. However, the flight activity of haematophagous insects can be greatly influenced by wind, and females of most mosquito species drastically reduce host-seeking flights when wind speeds are greater than about 3 km/h (0.83 m/s) [34]. Some mosquito species have been observed to fly close to the ground and to cling to the vegetation above certain wind speeds, e.g. *Aedes albopictus* [35]. In fact, wind speed affecting the mosquito flight behaviour is known to be species-specific (the wind speed threshold at which mosquitoes stop flying was reported to be between 3 km/h (0.83 m/s) for species in central Alaska and 29 km/h (8.06 m/s) for Canadian subarctic species [34]), and thus could serve as an indicator to describe the ecological niche of a species.

### Data

#### Species distribution data

**Fig. 1:**
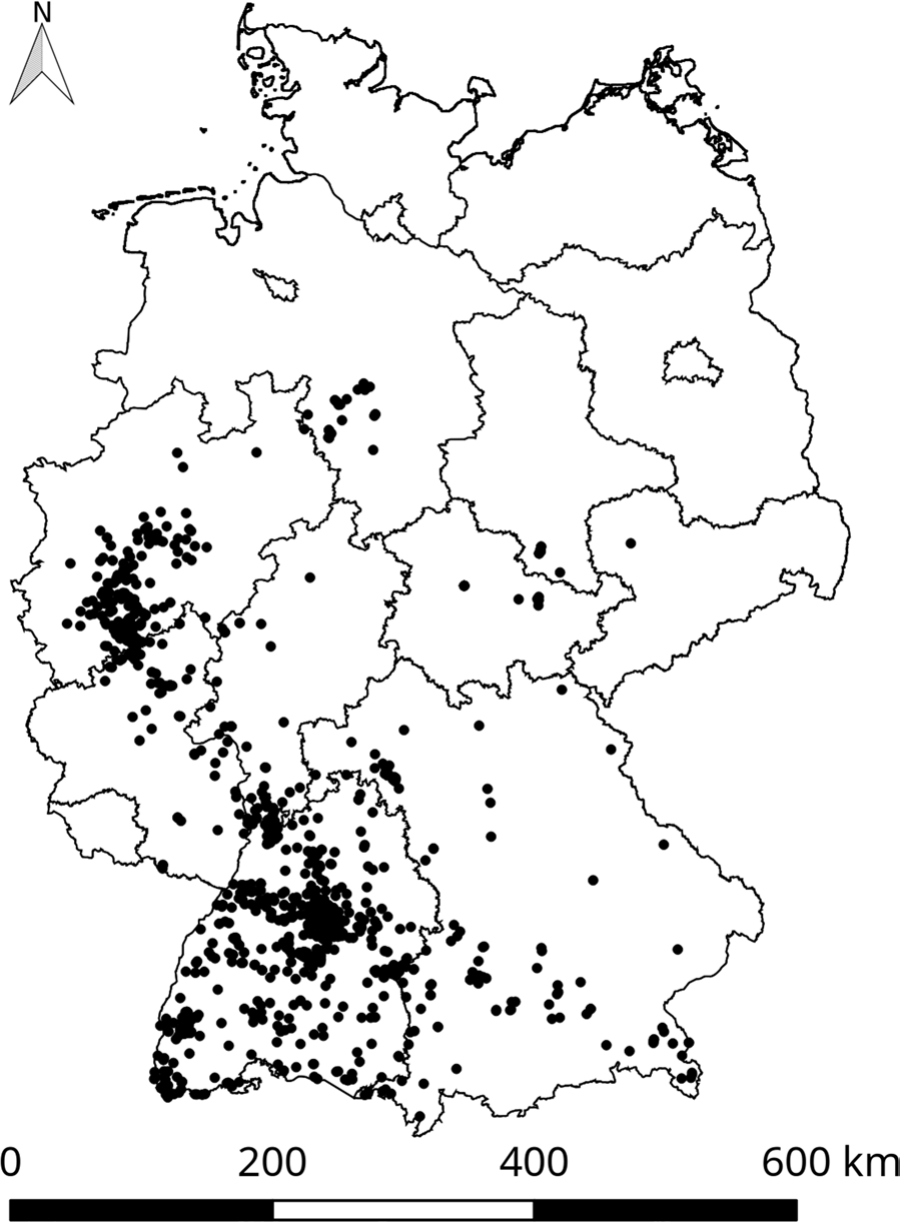
Collection sites of *Aedes japonicus japonicus* in Germany. The collection sites (*n* = 1110) are indicated as black dots and include samplings from the years 2012–2017. The geodata of Germany originate from the Bundesamt für Kartografie und Geodäsie [58]

Species collection data were relevant for the model to analyse and select environmental input data (to complete our understanding of the ecological dependencies) as well as to evaluate the model. They were derived from the German mosquito database “CULBASE” [36], which contains data from active and passive mosquito monitoring approaches. The passive monitoring data originate from the citizen science project “Mueckenatlas” [37] and the active monitoring data from inspections of regions and their adjacent areas from which invasive mosquito species were submitted. In the latter case, possible breeding habitats were screened for larvae, and traps were set up in some cases [37]. At the time of download (10 April 2018), the database included 1110 records of *Ae. j. japonicus* sampling sites from 2012–2017, 79% of them linked to passive monitoring. The distribution of the species in Germany regarding to this update is shown in Fig. 1.

#### Model input data and transformations

**Fig. 2:**
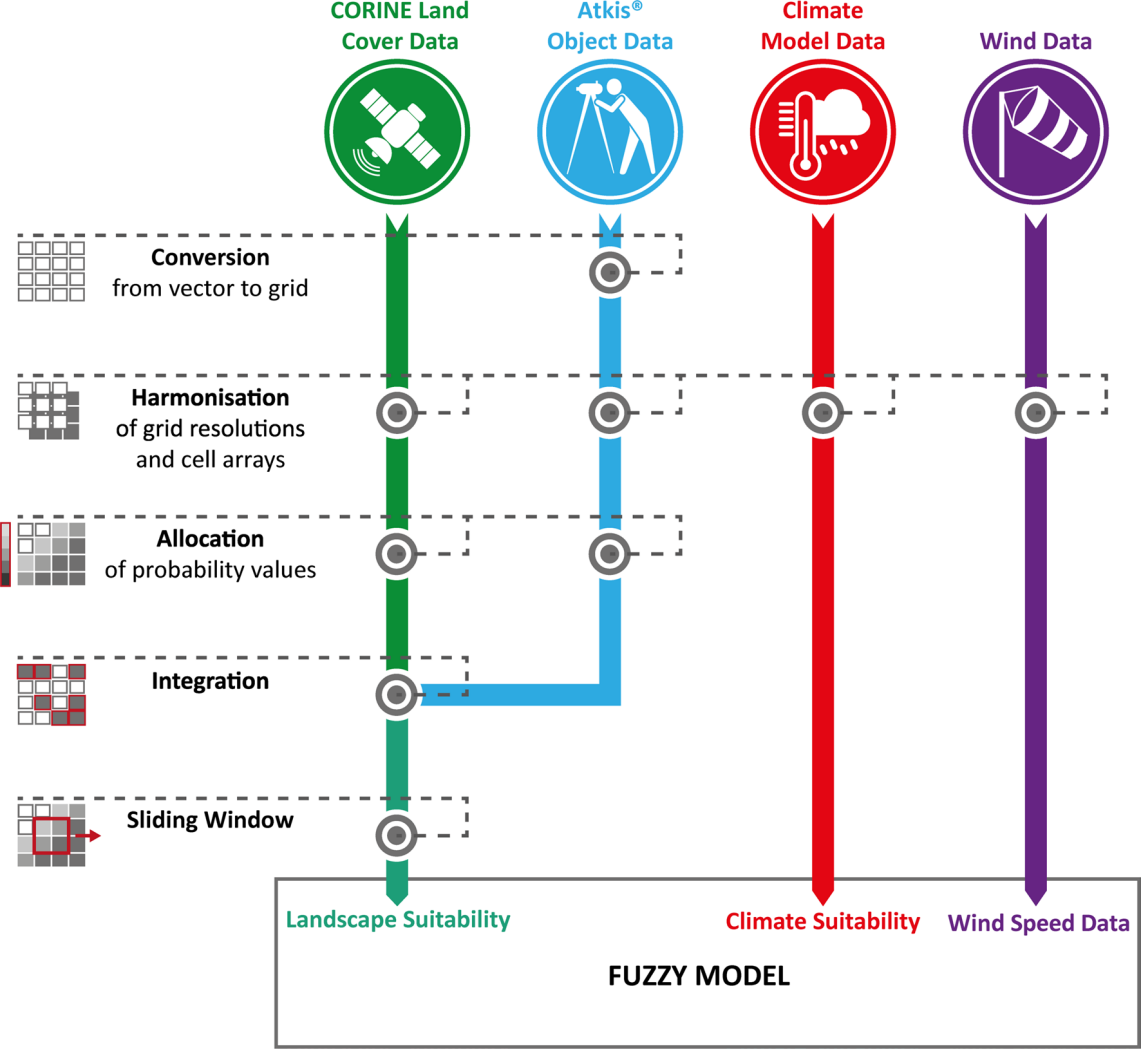
Input data of the fuzzy model and steps of pre-processing

The fuzzy model is based on three submodels that process appropriate geodata (climate data, land use data and wind data). To integrate all datasets into the model, they were pre-processed in several steps and harmonised in terms of file type, coordinate system, grid cell resolution and raster alignment (Fig. 2). Finally, the model input data were saved as grid files with a resolution of 100 × 100 m in the coordinate system DHDN Gauss-Kruger-Zone 3 (EPSG 31467). Data processing was done with the GDAL (1.11.3) library and Python (2.7). Additionally, the GRASS GIS tool “r.resample” was used to calculate the grid orientations.

#### Climate data

A dataset based on the approach by Früh et al. [17] was included into the fuzzy model. It defines the climate suitability of *Ae. j. japonicus* as values between zero and one, within Germany depending on climate variables according to Wieland et al. [38]. The underlying data have a resolution of 1 × 1 km and are derived from the German Weather Service [39]. They include the monthly precipitation sums of February, April and June, the autumn (average of September, October and November) drought index, the average monthly temperatures of September, October and December, and the average seasonal temperature of spring (average of March, April and May). The model is based on a support vector machine [40] as a training algorithm that distinguishes the climate niche of *Ae. j. japonicus* from the niche of three mosquito species native to Germany (*Aedes vexans*, *Aedes geniculatus* and *Anopheles daciae*).

To transform the climate model trained for the period 2012–2014 into a long-term climate model, a calibration was performed with mean climate data for the period of 1981–2010 (most recent international climate reference period) and an updated set of field collection data from 2012–2017. Ten percent of the collection data was reserved for k-fold cross validation. Additionally, we changed the data scaling before starting the training and calculated it by Eq. 1, with *x* being the input and *s*(*x*) being the scale(*x*):

1 $$s\left( x \right) = \frac{{x - \bar{x}}}{{\sigma^{2} }};\;\bar{x} = mean\left( x \right);\;\sigma^{2} = variance\left( x \right)$$

To forecast the future distribution of *Ae. j. japonicus* until 2050, we changed the input variables of the climate model according to the average of several IPCC climate change scenarios from the ATEAM project (HadCM3 SA1, HadCM3 SA2, HadCM3 SB1, HadCM3 SB2, CGCM2 SA2, CSIRO2 SA2 and PCM SA2) [41] and reapplied it. On average, summer temperature increased by 1.4 K, summer precipitation declined by 4%, and winter precipitation increased by 5%.

#### Land use data

Land use data were derived from satellite image interpretations from the CORINE Land Cover database [42] and the ATKIS vector data from the State Survey Authority [43]. The satellite image interpretations (Europe-wide dataset) had a resolution of 100 × 100 m and included 44 different land use types. From the ATKIS data, we extracted additional datasets that were relevant for the occurrence of the species. The vector data were gridded by assigning a 100 × 100 m grid cell from 51% fill level onwards. Considering the habitat requirements of the species, we assigned suitability values between zero (no suitability) and one (very good suitability) for each type of land use (Tables 1, 2).

**Table 1:** CORINE land use data. Suitability of land use types for the occurrence of *Aedes j. japonicus* with the attributes being derived from the CORINE Land Cover dataset

No.	CORINE land use category	Degree of suitability (from 0 to 1)	Percentual area of Germany	No.	CORINE land use category	Degree of suitability (from 0 to 1)	Percentual area of Germany
1	Continuous urban fabric	0.2	0.04	23	Broad-leaved forest	0.9	9.73
2	Discontinuous urban fabric	1	6.91	24	Coniferous forest	0.1	16.57
3	Industrial or commercial units	0.2	1.38	25	Mixed forest	0.8	4.08
4	Road and rail networks and associated land	0.5	0.06	26	Natural grasslands	0	0.42
5	Port areas	0.8	0.02	27	Moors and heathland	0	0.27
6	Airports	0.2	0.11	28	Sclerophyllous vegetation	–	–
7	Mineral extraction sites	0	0.20	29	Transitional woodland-shrub	0.6	0.63
8	Dump sites	0.2	0.04	30	Beaches, dunes, sands	0	0.03
9	Construction sites	0.3	0.01	31	Bare rocks	0.1	0.04
10	Green urban areas	1	0.20	32	Sparsely vegetated areas	0	0.03
11	Sport and leisure facilities	1	0.45	33	Burnt areas	–	–
12	Non-irrigated arable land	0	37.92	34	Glaciers and perpetual snow	0	3.63
13	Permanently irrigated land	–	–	35	Inland marshes	0	0.09
14	Rice fields	–	–	36	Peat bogs	0	0.21
15	Vineyards	0.1	0.35	37	Salt marshes	0	0.05
16	Fruit trees and berry plantations	0.3	0.42	38	Salines	–	–
17	Olive groves	–	–	39	Intertidal flats	0	0.05
18	Pastures	0.2	17.98	40	Water courses	0	0.21
19	Annual crops associated with permanent crops	–	–	41	Water bodies	0	0.89
20	Complex cultivation patterns	0.1	0.20	42	Coastal lagoons	0	0.05
21	Land principally occupied by agriculture, with significant areas of natural vegetation	0.2	0.27	43	Estuaries	0	0.03
22	Agro-forestry areas	–	–	44	Sea and ocean	0	0.06

**Table 2:** ATKIS land use data. Suitability of land use types for the occurrence of *Aedes j. japonicus* with the attributes being derived from the ATKIS dataset

ATKIS object category	Degree of suitability (from 0 to 1)	Percentual area of Germany
Cemeteries	1	0.11
Landfill sites	0.2	0.05
Garden centres	1	0.05
Gardens	1	0.37
Zoological gardens	1	0.01

Landscapes were classified as completely unsuitable if they either did not meet the known habitat requirements, as is the case with non-irrigated arable land and sparsely vegetated areas (because of their lack of shade and breeding sites), or with large open waters (which do not serve as breeding sites *inter alia* due to the presence of predators), or if they simply have not been reported to be appropriate habitats for *Ae. j. japonicus.* The latter applies to moors and heathland, beaches and dunes, glaciers and places with perpetual snow, marshes, and peat bogs.

Land use types and landscape structures that we assumed to be particularly suitable and that have been reported to be hot spot occurrence areas included: (i) broad-leaved and mixed forests (due to the availability of shade and resting sites); (ii) green urban areas; (iii) sport and leisure facilities; (iv) harbours (the last three mentioned due to their diverse habitat structure and the availability of breeding sites in the form of trash); (v) cemeteries (both due to the flower vase density and the abundance of flowers whose nectar serves as food, and because of its structural diversity including shady resting sites [44]); (vi) gardens (due to the availability of small water-filled containers such as rain barrels and flower pots and a similar landscape structure as cemeteries); (vii) zoological gardens (due to the high abundance of blood-feeding hosts, animal drinking stations that could function as breeding sites, and their diverse park-like landscapes [45]); and (viii) garden centres (where plenty of nectar and water-filled flower pots are available).

The CORINE and ATKIS suitability arrays were combined, overwriting the CORINE data with the higher-resolution, selected ATKIS data when available. We considered the interactions of neighbouring landscape elements by applying the sliding (or moving) window technology [46, 47]. The sliding window calculates the mean value for each grid cell and its surrounding cells within a certain distance. This leads to the result that highly rated cells in the neighbourhood of poorly rated cells become less highly rated and cells with originally low suitability can get upgraded by a very suitable neighbourhood. We tested sliding windows with sizes of 100 × 100 m to 1100 × 1100 m (100 m corresponds to one raster cell) at a stepwise enlargement by 200 m edge length each, because the window required an uneven pixel number. Then we intersected the outcome with the *Ae. j. japonicus* occurrence data and chose the window with 700 m edge length, which best described the natural distribution of the species, supposing that a successive increase of findings should be given with increasing degrees of landscape suitability. The resulting array, containing the suitability values from zero to one, was used as model input.

#### Wind data

Wind data were provided by the Climate Data Centre of the German Weather Service [39] and downloaded for the most recent international climate reference period of 1981–2010. They have a resolution of 200 × 200 m and rely on a statistical wind field model, which considers measurements 10 m above the ground as well as the geographical location, terrain and type of land use. The data are represented by continuous real values.

### Fuzzy rule-based modelling

**Fig. 3:**
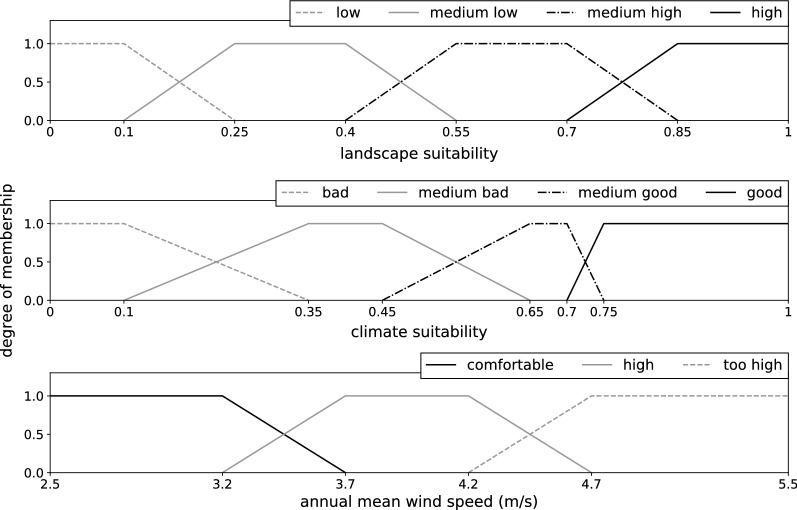
Membership functions of the fuzzy model

**Fig. 4:**
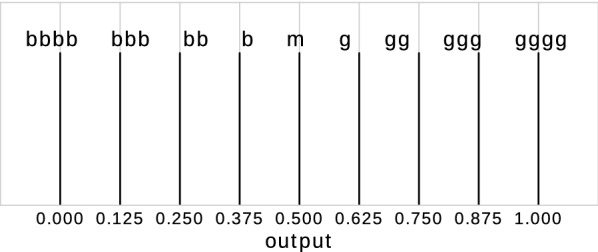
Output definitions of the fuzzy model

For each input dataset (climate suitability, wind speed and landscape suitability), we defined fuzzy sets by giving membership functions to linguistic terms, e.g. “wind speed is comfortable”, “wind speed is high” or “wind speed is too high” (Fig. 3). The membership functions were derived from statistical analyses with the *Ae. j. japonicus* occurrence data and the input raster maps of the model. We set nine thresholds to define occurrence probability values for the model output (Fig. 4). The value ‘bbbb’ represents the lowest occurrence probability (‘b’ for ‘bad’), ‘m’ a medium occurrence probability and ‘gggg’ the highest occurrence probability (‘g’ for ‘good’). In the next step, we defined the fuzzy rules (Table 3).

**Table 3:** Linguistic fuzzy rules

Premises			Conclusion
If the suitability of landscape is…	And if the wind speed is…	And if the suitability of climate is…	Then (linguistic)
low	comfortable	bad	bbbb
low	comfortable	medium bad	bbb
low	comfortable	medium good	bb
low	comfortable	good	b
low	high	bad	bbbb
low	high	medium bad	bbbb
low	high	medium good	bbb
low	high	good	bbb
low	too high	bad	bbbb
low	too high	medium bad	bbbb
low	too high	medium good	bbbb
low	too high	good	bbb
medium low	comfortable	bad	bb
medium low	comfortable	medium bad	b
medium low	comfortable	medium good	m
medium low	comfortable	good	g
medium low	high	bad	bbb
medium low	high	medium bad	bb
medium low	high	medium good	m
medium low	high	good	m
medium low	too high	bad	bbbb
medium low	too high	medium bad	bbbb
medium low	too high	medium good	bbb
medium low	too high	good	bbb
medium high	comfortable	bad	m
medium high	comfortable	medium bad	gg
medium high	comfortable	medium good	ggg
medium high	comfortable	good	gggg
medium high	high	bad	bb
medium high	high	medium bad	m
medium high	high	medium good	g
medium high	high	good	gg
medium high	too high	bad	bbbb
medium high	too high	medium bad	bbb
medium high	too high	medium good	bb
medium high	too high	good	bb
high	comfortable	bad	m
high	comfortable	medium bad	gg
high	comfortable	medium good	gggg
high	comfortable	good	gggg
high	high	bad	bb
high	high	medium bad	m
high	high	medium good	gg
high	high	good	ggg
high	too high	bad	bbbb
high	too high	medium bad	bbb
high	too high	medium good	bb
high	too high	good	bb

### Software and implementation

The tool Samt2Fuzzy from the software SAMT2 [48, 49] was used for implementing the fuzzy model. After applying the model, an output raster was created and saved as grid file using Python 2.7. The calculation time for one model application was 20.25 min on a computer with an Intel Xeon CPU E5-1620 v2 (3.70 GHz) processor under Ubuntu 16.04 (xenial). Detailed maps were generated with QGIS 2.14.

## Results

### Results of input models

**Fig. 5:**
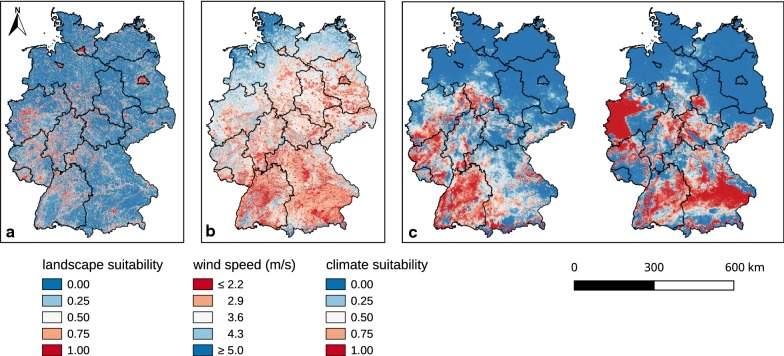
Model input data. Landscape suitability (**a**), mean wind speed in the period 1981–2010 (**b**), and climate suitability for the periods 1981–2010 (left) and 2021–2050 (right) (**c**). The geodata of Germany originate from the Bundesamt für Kartografie und Geodäsie [58]

Of the three input variables of the model (Fig. 5), two were based on submodels: landscape suitability and climate suitability. The climate model calculated for the actual weather conditions (1981–2010) reached an accuracy of 84.13% under 40-fold repeated trainings with a standard deviation of 1.22%. The intersections of the climate map with the occurrence points of *Ae. j. japonicus* yielded a median prediction value of 0.78 (mean 0.68).

**Fig. 6:**
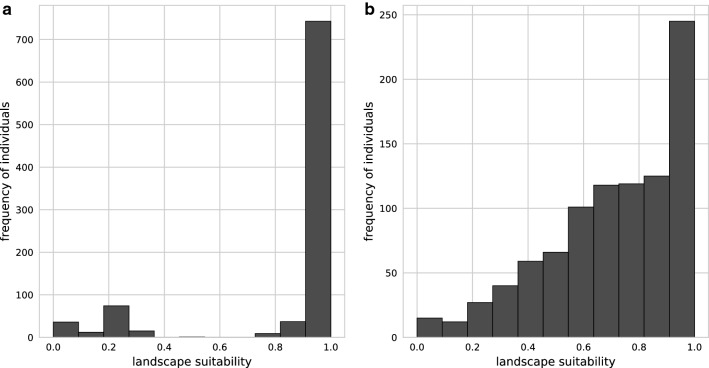
Procedure for selecting the pixel size of the sliding window. Occurrence probabilities at the collection sites of *Aedes japonicus japonicus* in Germany (1110 samplings), depending on the pre-processed land use data before applying the sliding window technique to the data (**a**) and after applying the technique with 700 m as a distance parameter (**b**)

**Fig. 7:**
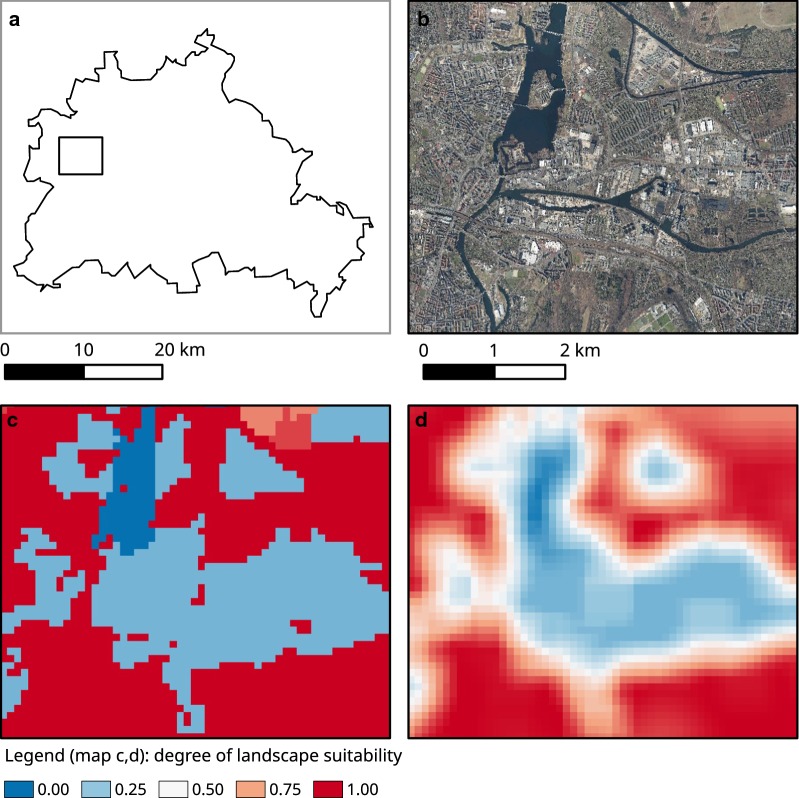
Illustration of the effect of the sliding window on the data. Image section from Germany-wide datasets of land use data in the area of Berlin. **a** Outline map. **b** Aerial photographs of the section (Senatsverwaltung für Stadtentwicklung und Wohnen Berlin, 2016). **c**, **d** Land use suitability maps based on the CORINE and ATKIS land use data before (**c**) and after (**d**) the application of the sliding window technique. These maps illustrate the model input factor “landscape suitability” on a large scale. A random area in the city of Berlin was chosen, where aerial photographs [59] can be freely utilised. The area mainly consists of continuous and discontinuous urban fabric, road and rail networks and associated land, green urban areas, gardens and water bodies. The outline map for the area of Berlin originates from geodata of the Bundesamt für Kartografie und Geodäsie [58]

For the landscape suitability model, the sliding window script was applied after assigning the suitability values for each land use type. The question arose how big the window had to be. An application for the number of seven pixels (corresponding to 700 m) turned out to be the most suitable distance measure. As shown in Fig. 6, the land use probabilities at the observed occurrence points of *Ae. j. japonicus* in Germany changed from a bimodal distribution to a unimodal left-skewed distribution, which better reproduces the real environmental conditions. When the number of pixels was increased to nine, the curve became bell-shaped and thus inappropriate for representing the relationship of landscape suitability and the number of species samplings. Figure 7 shows a section of the resulting land use dataset and how it developed by applying the sliding window technique. Intersecting the outcome of the landscape suitability model with the *Ae. j. japonicus* occurrence data gave a median prediction value of 0.75 (mean 0.71).

**Fig. 8:**
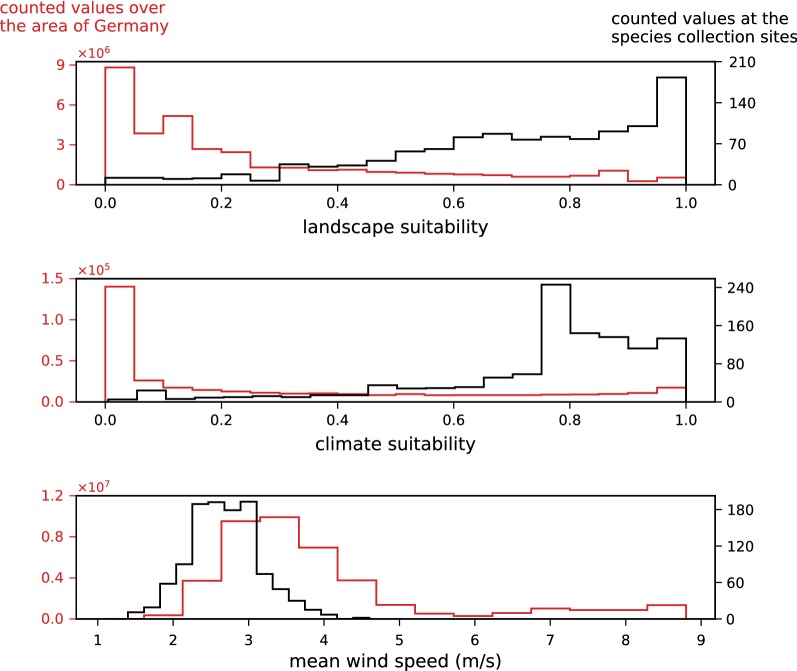
Analyses that helped define the fuzzy membership functions. Histograms showing for each input raster array (landscape suitability, climate suitability and mean wind speed) the distribution of values at the field collection sites (black line) compared to the distribution of values of the input raster arrays (red line)

**Fig. 9:**
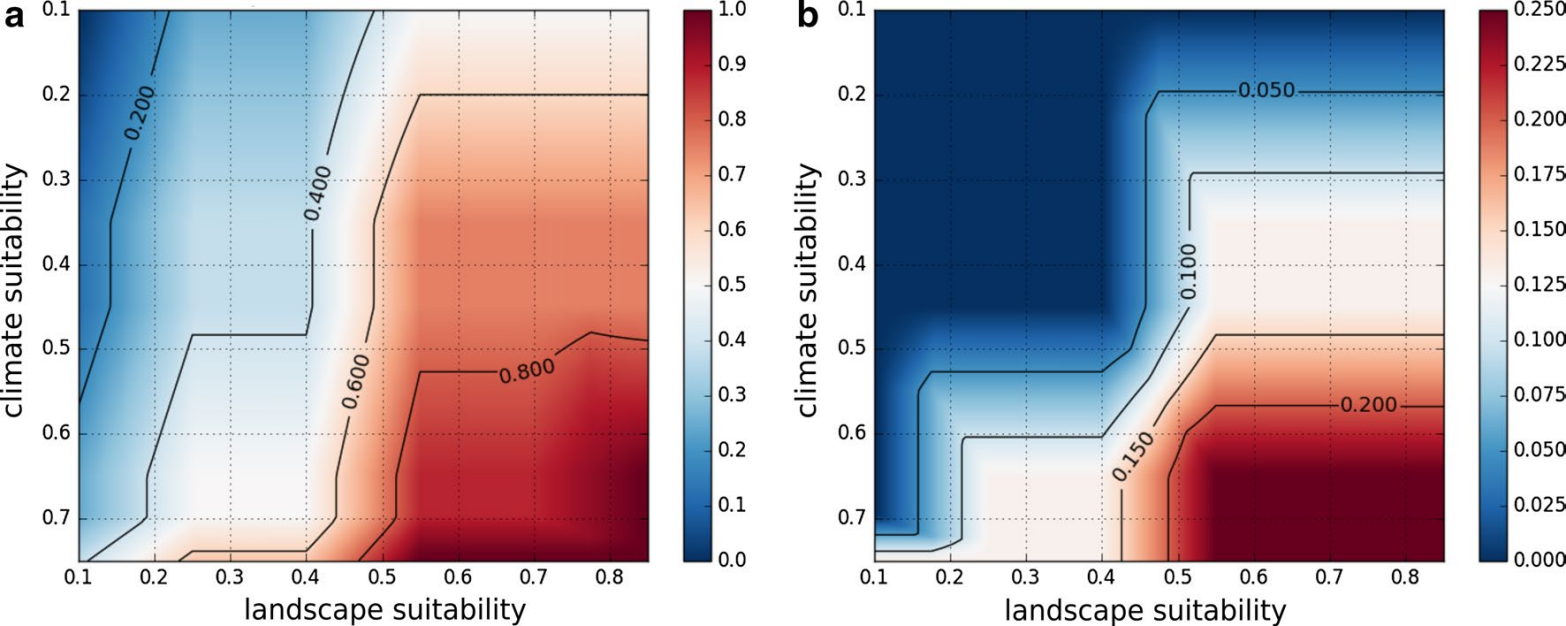
Illustration of the influence of wind speed in the model. Outputs of the fuzzy model according to wind speeds of 3 m/s (**a**) and 5 m/s (**b**). The different scales of both graphs illustrate the strong influence of the model input parameter ‘wind’

The membership functions of the fuzzy model defined for each input dataset (landscape suitability, climate suitability and mean annual wind speed) were derived from statistics that compare the distribution of the values over the entire area of Germany with the distributions at the sites where the mosquito species occurs (Fig. 8). For all parameters, the distribution curves at the sites of discovery clearly differed from the distributions over the entire area of Germany. Strikingly, *Ae. j. japonicus* was not shown to occur in regions of Germany characterised by wind speeds higher than 4.7 m/s. Therefore, special attention was paid to the input variable ‘wind’ when defining the fuzzy rules (Table 3). At average wind speeds of 3.7 m/s, the suitability for the occurrence of *Ae. j. japonicus* is already significantly reduced according to the model and at an average wind speed of 4.7 m/s, the model reduces habitat suitability to a maximum of 25%. Figure 9 displays the consequences of the fuzzy rule definitions on a metric scale.

### Results of the fuzzy model

**Fig. 10:**
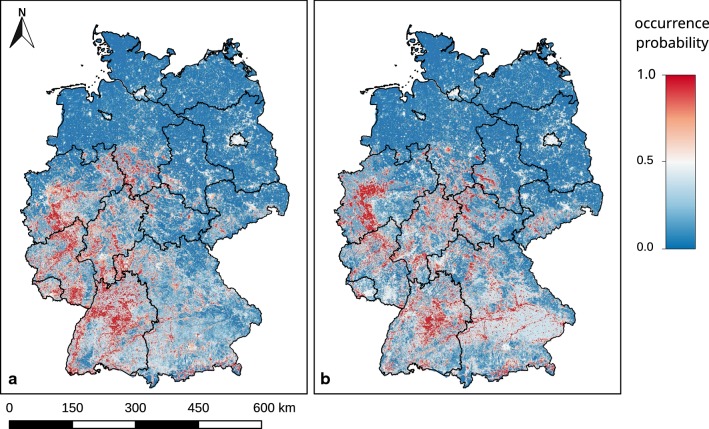
Results of model applications for current and future climate conditions. Occurrence probability of *Aedes japonicus japonicus*, depending on our fuzzy model approach applied for climate conditions of the period 1981–2010 (**a**) and the prediction of future climate conditions of the period 2021–2050 (**b**). Projection: Gauss-Krüger zone 3. The geodata of Germany originate from the Bundesamt für Kartografie und Geodäsie [58]

Our results (Fig. 10) provide a very detailed picture of how the Asian bush mosquito could spread under current and future climatic conditions. The prediction maps for both actual and future conditions reveal that urban areas are generally suitable for occurrence. Under current climatic conditions, the largest areas suitable for the mosquito are to be found in central to southwestern Germany. In southeastern Germany, appropriate areas will steadily expand under future climatic conditions. The coastal north, the generally more northern plains and parts of the alpine mountains in the south seem consistently unsuitable for the establishment of the species. Also remarkable is that highly suitable conditions are predicted for regions that are inappropriate according to the climate model input; at the same time, unsuitable conditions are predicted inside climatically suitable regions, e.g. in the southwestern part of the country.

### Evaluation of the fuzzy model

**Fig. 11:**
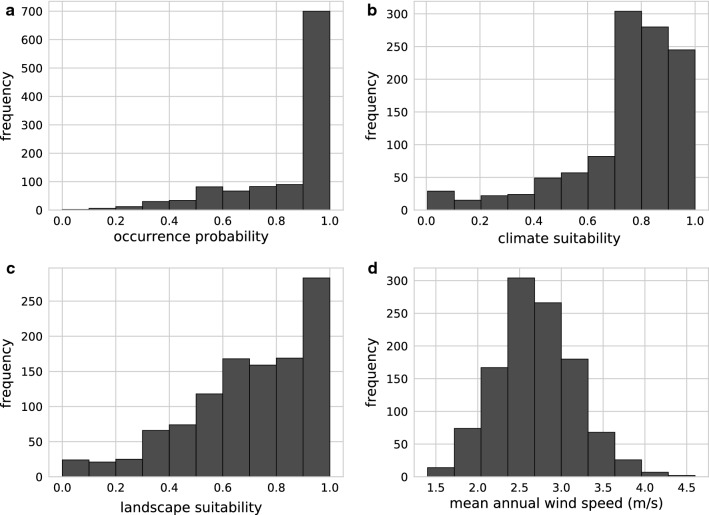
Model evaluation. Calculated probability and suitability for the occurrence of *Aedes japonicus japonicus* at the field sampling sites according to the fuzzy model application for 1981–2010 (**a**), the climate model for 1981–2010 (**b**) and the landscape model (**c**). The wind histogram (**d**) demonstrates the mean annual wind speeds for 1981–2010 at the sampling sites. The species sampling data are from the years 2012–2017 (database update 10 April 2018, number of collections = 1110)

**Fig. 12:**
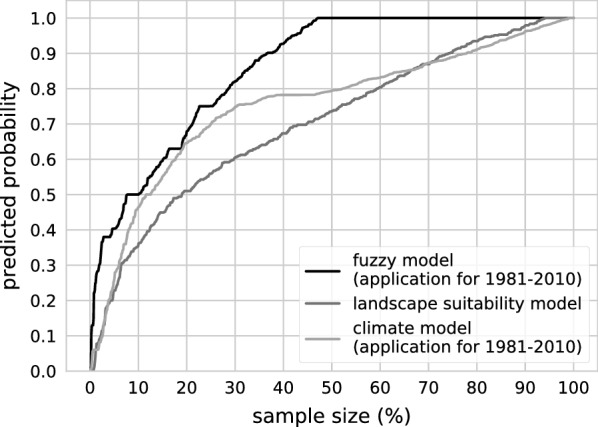
Evaluation of the fuzzy model in comparison to the underlying models. Cumulative gain chart showing the calculated occurrence probabilities at *Aedes japonicus japonicus* field sampling sites. The occurrence probabilities are derived from the application of the fuzzy model for the period 1981–2010 (black line) and, for comparison, from the fuzzy model inputs “landscape suitability” (dark grey line) and “climate suitability”, depending on the application of the climate model for the same time period (light grey line). The species sampling data are from the years 2012–2017 (database update 10 April 2018, number of collections = 1110)

In the application of the fuzzy model to the climatically suitable areas of the years 1981–2010, the predictive values, ranging from 0 to 1.0, at the validation points (*n* = 1110) show a dominance of 1.0 values and a strongly left-skewed distribution (Fig. 11). Comparing the fit values of the fuzzy model with those of the input models of landscape suitability and climate suitability (Fig. 12), it becomes clear that the fuzzy model explains the occurrence of the species significantly better than the input models. The ‘exactness’ after Früh et al. [17] (average prediction value at all validation points) is 0.86. Small occurrence probabilities with a maximum value of 0.5 still accounted for 10% of the predictive values at the validation points (Fig. 12), yielding a model selectivity [17] of 0.85 (‘selectivity’ considers the threshold prediction value at 10% of the lowest predictions at the species collection sites, and reflects the percentage size of the area of Germany that remains unsuitable at this threshold.).

## Discussion

### Model validation

The fuzzy model scored very well in the validation regarding the analysis of the species collection sites. Compared to the climate models based on combinations of different machine learning methods [17], the fuzzy model achieved a significantly higher value of exactness (0.86 compared to 0.63–0.65) and selectivity (0.85 compared to 0.63–0.67), while providing a ten times higher model resolution (only the three best results of the mentioned study were used for comparison). The better performance of our model can be partly explained by the calibration of the climate model, with a larger set of occurrence data being used for training (data for the years 2015–2017 were additionally used, but not those from 2011 which were only few). However, the calibrated climate model scored only slightly better in the validation, the exactness improved by 5–7% while the data resolution remained unchanged.

We did not calculate the standard model quality parameter of AUC (area under the curve [50]) due to the lack of true absence data in our study. We also decided to not generate any pseudo-absence data for evaluating the model, as such data have a high degree of uncertainty caused by the methodology of collecting the model species [50]. In our case, the collection data are especially unsuitable for identifying unsuitable land use types, as 79% of the validation data originate from the citizen science project “Mueckenatlas”, hence the collections are mostly derived from densely populated regions. By contrast, data from the active collections came from deliberately selected sites, in particular cemeteries and private gardens. Forested and agricultural regions are, however, underrepresented in the collection data.

### Advantages and disadvantages of the fuzzy model approach

The dependence of the *Ae. j. japonicus* findings on the monitoring method was a strong argument for using a modelling technique that can be directly controlled by the modeller and that is not based on machine learning algorithms. Therefore, we chose the method of fuzzy modelling where biological expert knowledge and field observations could be integrated into the model. The logical approach made the model robust against the discovery of dependencies not existent in reality (e.g. significantly more individuals of the species occur in less densely populated areas than the monitoring data confirm, but this has no effect on the model). The approach, however, also contained sources of error because we were dependent on the availability of biological knowledge about this species which is still incomplete. It is also possible that we misjudged the importance of certain environmental requirements of *Ae. j. japonicus* or, based on the areas investigated, developed a subjective impression of possible landscape suitability, which was incorrect or not applicable throughout Germany (see subsection “Input data” below).

### Input data

The process of creating the dataset of landscape suitability was challenging, mainly because some ecological characteristics of *Ae. j. japonicus* have not yet been determined. This led to uncertainties in the allocation of suitability values for some forms of land use (Tables 1, 2). Major uncertainties concerned human settlements and coniferous forests. Regarding human settlements, we have not differentiated between urban and rural settlements. This differs from studies in which significantly more individuals of *Ae. j. japonicus* were found in rural areas compared to urban areas [22, 51]. However, this observation cannot be confirmed for Germany. A large number of collection data linked to the citizen science project “Mueckenatlas” was obtained from both rural and urban areas; only centres of larger cities seemed hardly populated. Gardens and discontinuous urban fabrics therefore received a suitability value of 1.0 on a scale between 0 and 1, while a low value of 0.2 was allocated to the category of continuous urban fabric. A residential dataset specifically for rural areas was not integrated into the landscape model.

Also for coniferous forests, little information is available about the habitat suitability for *Ae. j. japonicus*. The biologists among us had different experiences. For example, some observed little to no larvae of the species in coniferous forests within their distribution areas in Germany. If containers (small clay pots) were made available, however, these were colonised. They also reported that larvae usually do not occur in flower vases in populated cemeteries under certain coniferous species, while they can be found under deciduous trees. This is probably because substances in the needles of various species (terpenes and oil) can be detrimental for larvae and pupae of mosquitoes [52, 53]. Therefore, and also because it is unusual that conifers provide cavities that can fill with water compared to deciduous trees [54, 55], we have assigned a low suitability value (0.2) to coniferous forests.

The distance at which land use types influence each other in terms of the potential occurrence of the species has also not yet been widely investigated by biologists. In repeated applications of the sliding window (for the creation of the landscape suitability input map) with different window sizes, the most plausible results were shown for a size of 700 × 700 m, which corresponds to a radius of around 350 m when looking at the central pixel of the window. Our finding roughly corresponds to the results of flight distance studies of mosquitoes, as summarised by Verdonschot and Besse-Lototskaya [56]. These authors showed that, although the maximum flight distance of an individual of the species can be up to 1600 m, the mean flight distance, measured by mark-recapture experiments, within the genus *Aedes* is only 89 m with a standard deviation of 50 m (to our knowledge, there is no information about the mean flight distance of *Ae. j. japonicus*). Also of interest are calculations of the percentual reduction of the number of mosquitoes with increasing distance when an inhospitable buffer zone is established. *Aedes albopictus*, which is also a container-breeding species in settlements and has similar host preferences, would be reduced by 99% for a 617 m wide barrier, 90% for a 347 m wide barrier and 70% for a 97 m wide barrier [56].

Another reason why the creation of the landscape suitability dataset was challenging was due to difficulties in data acquisition. It is possible that the age structure of deciduous trees could also be taken into account into the landscape model, since young deciduous trees have fewer tree holes than older trees. However, since such a dataset is not available for Germany, we were not able to evaluate this aspect and integrate it into the landscape model. Another problem caused by the availability of geodata was the combination of berry fruit and fruit tree plantations. This category of land use was derived from the CORINE dataset and is problematic, as fruit tree plantations are probably well suited and berry shrub plantations clearly poorly suited habitats for *Ae. j. japonicus*. We have given this category a rather low value of landscape suitability (0.3 within a range of 0 to 1) as there are yet no particular occurrence reports of *Ae. j. japonicus* from within fruit tree plantations (where pesticide application might also have a negative effect on the development of mosquitoes). With an additional effort, the two types of land use could be separated from each other to improve the model, e.g. by satellite image analysis. However, fruit tree and berry plantations cover less than 0.5% of the total area of Germany, so the unfavourable combination of both forms of land use into one category is not expected to reduce the quality of the model significantly.

Wind as a model input is an interesting novelty compared to previously published climate models for the occurrence of *Ae. j. japonicus* [7, 15–17]. This factor significantly improves the model. However, it is also a parameter that is dependent on land use. Wind data for Germany are also not exclusively based on measurements but partly on a model that takes into account land use as well as terrain elevation and geographical location. The data relate to 10 m above ground level, but a wind speed map related to a maximum of 5 m above ground would be preferable for our purposes.

The climate model that served as input for this approach shows similar results to other climate models for the occurrence of *Ae. j. japonicus* in Germany under current climatic conditions [15, 16]. Under future conditions, however, the results from [15] differ significantly from ours: a general reduction of suitable areas is predicted, while our forecast indicates a continuing high availability of suitable areas in Germany, only with partially shifted central areas. The difference is probably due to the use of different climate variables and training data of *Ae. j. japonicus* as well as to the application of different modelling approaches. Generally, the estimation of the effects of climate change on the potential distribution of the species is very vague in all approaches, since no regional effects have been considered and the forecasts of precipitation development in Germany vary considerably. Precipitation, however, is of particular importance for container-breeding mosquito species.

Concerning the result of the fuzzy model for the probable future conditions of the years 2021–2050 (Fig. 10b) one has to be aware that the aspect of land use change, which in turn might have an influence on wind conditions, is not considered.

### Fuzzy rules

The model applications show highly suitable occurrence areas in climatically unsuitable regions, especially in densely populated areas. This is due to the fuzzy rules we have established based on the assumption that an unsuitable climate can partially compensate for a very suitable form of land use. We founded this assumption on the fact that the climate model, which served as input to the fuzzy model, had a high proportion of precipitation variables on all climate parameters, and the climate model classified areas with low precipitation as unsuitable. This is correct for calculating the climatically suitable regions for *Ae. j. japonicus* in Germany, as the species is relatively tolerant to different temperature conditions considering its ecological adaptations to cold regions as well as its occurrence in subtropical to tropical regions (Florida [6], Hawaii [51]) and the Mediterranean region (Spain [10]), in addition to its predominance in cool temperate zones.

However, certain types of land use can compensate for the lack of precipitation, e.g. in residential areas and gardens it can be assumed that people regularly refill flower pots, that rain water barrels and wells are available or that there are irrigation systems. In forested areas, cool air and limited insolation can reduce evaporation, which means that the water in tree cavities probably lasts longer than in other water containers.

Conversely, we assumed that an unsuitable land use type can only marginally be outweighed by a suitable climate in our model, as the absence of certain habitat characteristics make the occurrence of the species considerably more unlikely. For example, there are no breeding and shady resting sites on pastures, which cannot be compensated for by an appropriate climate.

The fuzzy model could be further improved by training procedures as for example applied in Wieland and Mirschel [57]. Another important step would be to build a model that considers the propagation paths and invasion speeds of *Ae. j. japonicus*. Simulation applications are presently being planned for this purpose.

## Conclusions

The paper introduces a nested approach to model the habitat suitability of invasive mosquito species (here *Aedes japonicus japonicus* in Germany). The first step of the approach is to model the habitat suitability with respect to climate variables using machine learning. The second step is the development of a model that considers regional influences such as land use and the availability of specific landscape elements. For this purpose, the integration of expert knowledge has proven to be useful. In a final step, these models and any further relevant data can be logically combined by means of fuzzy modelling. The nested approach has proven to be very effective in this study. We were able to generate potential distribution maps with a high prediction accuracy and spatial resolution of 100 × 100 m, which could serve as a basis for the conceptual design of control measures in the event of a disease outbreak mainly caused by the vector activity of *Ae. j. japonicus*. The combination of all parameters could better explain the distribution pattern of the species in Germany than the individual models (climate or landscape only) and data (wind). All model input data, scripts and software are open-source and freely available, so the model can easily be applied to other countries or, more generally, to other species, especially, but not exclusively, within the family of Culicidae.

## Abbreviation

*Ae. j. japonicus*: *Aedes japonicus japonicus*
